# A tutorial on Bayesian multi-model linear regression with BAS and JASP

**DOI:** 10.3758/s13428-021-01552-2

**Published:** 2021-04-09

**Authors:** Don van den Bergh, Merlise A. Clyde, Akash R. Komarlu Narendra Gupta, Tim de Jong, Quentin F. Gronau, Maarten Marsman, Alexander Ly, Eric-Jan Wagenmakers

**Affiliations:** 1 Department of Psychological Methods, University of Amsterdam, Postbus 15906, 1001 NK Amsterdam, The Netherlands; 2 Duke University, Durham, NC USA; 3 Centrum Wiskunde & Informatica, Amsterdam, The Netherlands

**Keywords:** Bayesian inference, Bayesian model averaging, Linear regression

## Abstract

Linear regression analyses commonly involve two consecutive stages of statistical inquiry. In the first stage, a single ‘best’ model is defined by a specific selection of relevant predictors; in the second stage, the regression coefficients of the winning model are used for prediction and for inference concerning the importance of the predictors. However, such second-stage inference ignores the model uncertainty from the first stage, resulting in overconfident parameter estimates that generalize poorly. These drawbacks can be overcome by model averaging, a technique that retains all models for inference, weighting each model’s contribution by its posterior probability. Although conceptually straightforward, model averaging is rarely used in applied research, possibly due to the lack of easily accessible software. To bridge the gap between theory and practice, we provide a tutorial on linear regression using Bayesian model averaging in JASP, based on the BAS package in R. Firstly, we provide theoretical background on linear regression, Bayesian inference, and Bayesian model averaging. Secondly, we demonstrate the method on an example data set from the World Happiness Report. Lastly, we discuss limitations of model averaging and directions for dealing with violations of model assumptions.

Linear regression is a standard statistical procedure in which one continuous variable (known as the dependent, outcome, or criterion variable) is being accounted for by a set of continuous predictor variables (also known as independent variables, covariates, or predictors). For concreteness, consider a researcher who is interested in predicting people’s happiness using a number of country-specific demographic indicators such as Gross Domestic Product (GDP), public safety, life expectancy, and many others. When all available predictors are included in the regression equation, the resulting model will generally overfit the data, the estimates of the regression coefficients will be unreliable, and the results will generalize poorly to other data sets (e.g., Myung 2000). Therefore, most regression analyses start by reducing the set of initial predictors to a relevant subset. The challenge of identifying a good subset is known as the model selection or variable selection problem. For instance, a variable selection procedure may suggest that only wealth and life expectancy are needed to predict happiness. Once the relevant subset has been identified, the associated regression model can be used to assess the magnitude of the relations between the criterion variable and the selected subset of predictors (e.g., how much we expect happiness to change per unit of change in wealth).

Although common practice, the two-step procedure has been known to be problematic for over 25 years (e.g., Hurvich and Tsai 1990; Miller 1990). Specifically, the second step in the two-step procedure ignores the uncertainty associated with the first step, that is, the uncertainty with which the model of interest (i.e., the subset of predictors) was obtained. Consequently, inference from two-step methods has been shown to be misleading (Draper, 1995) and result in overconfident parameter estimates and biased inference (Burnham & Anderson, 2003, Ch. 1.7). As summarized by (Claeskens & Hjort, 2008, Ch 7.4, p. 199):

“*‘Standard practice’ has apparently become to use a model selection technique to find a model, after which this part of the analysis is conveniently forgotten, and inference is carried out as if the selected model had been given a priori. This leads to too optimistic tests and confidence intervals, and generally to biased inference statements.*” (italics in original)

The principled alternative to the two-step procedure is multi-model inference. Instead of settling, perhaps prematurely, on a single model for inference, multi-model inference retains all models and calculates for each model a weight that indicates the degree to which the data support that model. These weights are usually a function of the posterior model probabilities, which represent the relative probability in favor of each model after the data are observed (Raftery, Madigan, & Hoeting, 1997; Hoeting, Madigan, Raftery, & Volinsky, 1999). At the same time that the model weights are being obtained, parameter estimates are calculated for each model. Then, instead of basing all of our inferences on a single model, we can take into account all of the models simultaneously. For example, in order to predict a set of new observations we first generate predictions from the individual models and then average these predictions using the posterior model probabilities as weights. This ensures our final prediction for new observations reflects our uncertainty across the entire model space (Claeskens & Hjort, 2008, Ch. 7). In other words, multi-model inference accomplishes variable selection and parameter estimation simultaneously instead of sequentially.

Despite the advantages of multi-model inference (e.g., Burnham, Anderson, & Huyvaert, 2011; Hinne, Gronau, van den Bergh, & Wagenmakers, 2020; Hoeting et al. 1999) and its successes in fields such as machine learning (Breiman, 2001), cosmology (Trotta, 2008), and climate prediction (Tebaldi & Knutti, 2007), the procedure has been applied only rarely in psychology (but see e.g., kaplan and Lee 2016; Gronau et al. 2017). The lack of multi-model inference in psychological science may be due in part to the perceived lack of user-friendly software that executes the analysis, as well as a dearth of tutorial-style explanations that allow psychologists to interpret the results of multi-model inference.

This aim of this paper is to bridge the gap between theory and practice by providing a tutorial on Bayesian multi-model inference, with an emphasis on user-friendly software to execute the analysis. First, we briefly provide theoretical background on linear regression, Bayesian inference, and Bayesian multi-model inference. Next we demonstrate the method in action using the BAS R package (Clyde, 2018) as implemented in JASP (JASP Team, 2020), an open source software program with a graphical user interface. The paper concludes with a summary and a discussion about pitfalls of regression modeling.

## Theoretical background

Before demonstrating Bayesian multi-model linear regression for a concrete data set we first introduce some basic theory. The impatient reader may skip this section. Below we first introduce linear regression, its assumptions, and the most common measure of effect size, *R*^2^. We then briefly describe Bayesian inference and finally introduce multi-model inference.

### Linear regression

The most common definition of multiple regression is:

1 $$ \begin{array}{@{}rcl@{}} y_{i} &= \beta_{0} + \beta_{1} x_{i1} + \beta_{2} x_{i2} + {\dots} + \beta_{p} x_{ip} + \epsilon_{i}, \end{array} $$

where *i* refers to the scores of the *i*^th^ subject and *p* to the total number of predictors. The intercept is represented by *β*_0_, and the linear effects between criterion and predictor variables are given by the regression coefficients *β*_1_, $\dots $, *β*_*p*_. The residuals (*𝜖*_*i*_) are assumed to be normally distributed with mean 0 and unknown variance *σ*^2^. The predictors $\left ( {x}_{1},  {x}_{2}, \dots ,  {x}_{p}\right )$ are usually centered (i.e., modeled with their mean subtracted, for example $\beta _{1} \left (x_{i1} - \overline {x}_{1}\right )$) so that inference about the intercept is independent of which predictors are included in the model. We will refer to collections of parameters or data points (vectors) using bold notation (e.g., *y* denotes *y*_1_, *y*_2_, $\dots $, *y*_*n*_).

From the definition of linear regression, it is evident that the model space can be enormous; consequently, linear regression presents a multi-model problem. With *p* predictors, $ {x}_{1}, \dots ,  {x}_{p}$, each of which can be included or excluded from the model, the total model space consists of 2^*p*^ members (e.g., with 10 predictors, there are 1024 different models to consider; with 15 predictors, the space grows to 32,768 models). If interaction effects are considered, the model space grows even more rapidly.

**Fig. 1:**
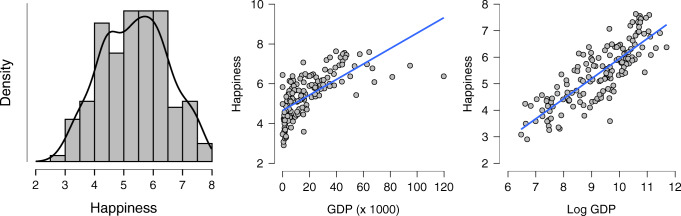
Example of a non-linear relationship between Happiness and Wealth, measured in terms of GDP. The left panel shows the density estimate for Happiness, the middle and right panel relate Happiness (*y*-axis) to GDP and log-transformed GDP (*x*-axes), respectively

Results from a linear regression analysis can be misleading if its assumptions are violated. The key assumption of linear regression is that the residuals are normally distributed. Introductory texts often mention other assumptions, but these assumptions generally concern specific violations of normality. We recommend three visual checks for assessing normality. As the name linear regression suggests, the relation between the predictor variables and the criterion variable should be approximately linear. Therefore, the first visual check we recommend is examining a scatter plot of the criterion and predictor variables. For example, suppose we wish to predict Happiness using Wealth. We might observe that the distribution of Wealth is right skewed and that the relation between Happiness and Wealth is non-linear. Such deviations from linearity can be corrected using, for instance, a log-transformation. Note that because of such transformations, linear regression analyses can detect more than just linear trends. The relation between Happiness and Wealth is shown in Fig. 1.

**Fig. 2:**
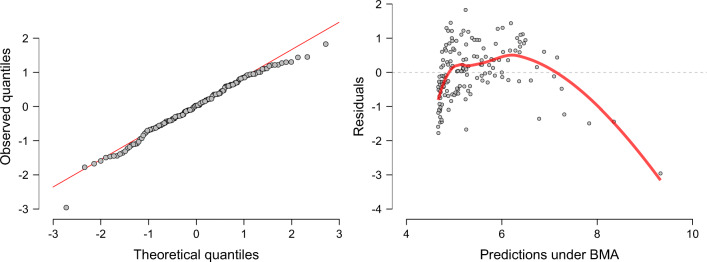
Assumptions checks for a linear regression where Happiness is predicted from Wealth, measured in terms of GDP. The left panel shows a Q-Q plot of the theoretical quantiles expected under a normal distribution (*x*-axis) against the quantiles of the observed residuals obtained from Bayesian Model Averaging (BMA; *y*-axis). The residuals appear approximately normally distributed. The right panel plots the predictions under BMA (*x*-axis) against the residuals (*y*-axis). Figures from JASP

Second, we recommend examining a Q/¯Q plot to assess the normality of the residuals. A Q/¯Q plot shows the quantiles of a theoretical normal distribution against the observed quantiles of the residuals. If the observed residuals are approximately normal, then all points in the plot fall approximately on a straight line. However, not all deviations from normality are easy to detect in a Q/¯Q plot. For instance, a Q/¯Q plot does not clearly show if the residuals are heteroscedastic, that is, the variance of the residuals is not constant across predictions. Therefore, our third recommendation is to plot a model’s predictions against a model’s residuals, which is a common visualization to assess heteroscedasticity and nonlinearity. To illustrate, we again predict Happiness with Wealth as measured in GPD. The left panel of Fig. 2 shows a Q/¯Q plot of theoretical against observed residuals and indicates little deviation from normality. However, the right panel of Fig. 2 visualizes the model’s predictions against the model’s residuals and suggests that the variance of the prediction error depends on the model’s predictions. For example, the residuals for a prediction of 5 are much more spread out than the residuals for a prediction of 6. In the right panel, the red line is a smoothed estimate of the mean at each point, obtained with local polynomial regression (Cleveland, Grosse, & Shyu, 1992). If the red line were horizontal with intercept zero, this would indicate that there is no structure left in the residuals that could be captured by the model (e.g., with interaction effects or higher-order polynomial terms).

However, here the red line varies as a function of the predictions, most likely because the relation between predictor and criterion is non-linear. Furthermore, the variance of the residuals differs across the predictions. This indicates that the residuals are heteroscedastic.

A linear regression of Happiness predicted by log-transformed GDP yields residuals that are better in agreement with the assumptions of linear regression (see Appendix B, Fig. 13).

After applying the regression model of interest and having confirmed that the assumptions are not badly violated, it is recommended to assess model fit. Model fit indices provide an idea about how well the model describes the data. Among the many model fit indices, the most common is the coefficient of determination *R*^2^ (Olive, 2017, p. 31), defined as

2 $$ \begin{array}{@{}rcl@{}} R^{2}_{\mathcal{M}_{j}} &= \text{Cor}\left( {y}, \hat{{y}} \mid \mathcal{M}_{j}\right)^{2}. \end{array} $$

$R^{2}_{{\mathscr{M}}_{j}}$ is the proportion of variance of the criterion variable *y* that is explained by model ${\mathscr{M}}_{j}$. The explained variance is computed by squaring the sample correlation between the observations *y* and the predictions $\hat { {y}}$ of ${\mathscr{M}}_{j}$.

Usually, the term ${\mathscr{M}}_{j}$ is omitted for brevity. Since *R*^2^ is the square of a correlation it always lies between 0 (poor model fit) and 1 (perfect model fit). It should be stressed that *R*^2^ is *not* a good measure for model comparison because it does not penalize models for complexity: when additional predictors are added to a model, *R*^2^ can only increase. Therefore, *R*^2^ will always favor the most complex model. However, the most complex model often fits the data too well, in the sense that idiosyncratic noise is misperceived to be systematic structure. In other words, complex models are prone to overfit the data (e.g., Hastie, Tibshirani, and Friedman, 2001, Ch. 7; Myung and Pitt, 1997; Vandekerckhove, Matzke, and Wagenmakers, 2015). Because models that overfit the data treat irreproducible noise as if it were reproducible signal, predictive performance for new data suffers. Altogether,

this makes *R*^2^ unsuitable for model selection, unless the competing

models have the same number of predictors.

### Bayesian inference

The next sections provide a brief introduction to Bayesian statistics. For accessible, in-depth tutorials and an overview of the literature we recommend the recent special issue in *Psychonomic Bulletin & Review* (Joachim Vandekerckhove, Rouder, & Kruschke, 2018).

#### Bayesian parameter estimation

Given a specific model ${\mathscr{M}}_{j}$ –in regression, a particular subset of predictors– we start a Bayesian analysis by defining prior beliefs about possible values for the parameters (e.g., the regression coefficients). This belief is represented as a probability distribution; ranges of likely values have more prior probability and ranges of less likely values have less prior probability.

As soon as data $\mathcal {D}$ are observed, Bayes’ theorem (Eq. 3) can be used to update the prior distribution to a posterior distribution:

3 $$ \begin{array}{@{}rcl@{}} \underbrace{p({\beta} \mid \mathcal{D} , \mathcal{M}_{j})}_{\text{Posterior}} &= \overbrace{p({\beta}\mid \mathcal{M}_{j})}^{\text{Prior}} \enspace \times \enspace \underbrace{\overbrace{ \frac{p(\mathcal{D} \mid {\beta}, \mathcal{M}_{j})}{p(\mathcal{D} \mid \mathcal{M}_{j})} }^{\text{Likelihood}}}_{\substack{\text{Marginal}\\ \text{Likelihood}}}. \end{array} $$

**Fig. 3:**
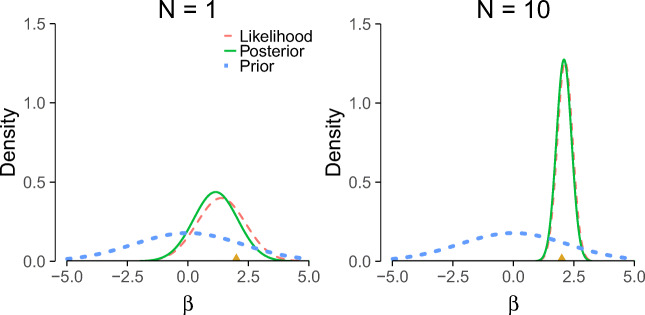
Illustration of Bayesian updating using Bayes’ theorem for a single observation (left panel) and ten observations (right panel). The ‘true’ value is 2 and is indicated by the gold triangle on the *x*-axes. Note that (1) the posterior depends less on the prior as more data are observed; (2) the variance (width) of the posterior decreases with sample size. In other words, we become more certain of our estimates as we observe more data. In the right panel, the likelihood was normalized for illustrative purposes. This example is based on normally distributed data with unknown mean and known variance (for derivations, see Murphy, 2007)

Equation 3 shows that our prior beliefs are adjusted to posterior beliefs through an updating factor that involves the likelihood (i.e., predictive performance for specific values for *β*) and the marginal likelihood (i.e., predictive performance across all values for *β*): values for *β* that predicted the data better than average receive a boost in plausibility, whereas values of *β* that predicted the data worse than average suffer a decline (e.g., Wagenmakers, Morey, & Lee, 2016). Equation 3 also shows that the posterior distribution is a compromise between the prior distribution (i.e, our background knowledge) and the data (i.e., the updating factor). The updating process is visualized in Fig. 3. Note that the impact of the prior on the posterior becomes less pronounced when sample size increases. In large samples, the posterior is often dominated by the likelihood and the posterior is practically independent of the prior (Wrinch & Jeffreys, 1919). In addition, with more data the posterior distribution becomes increasingly peaked, reflecting the increased certainty about the value of the parameters.

#### Bayesian model selection

The parameter estimation procedure provides us with posterior distributions for parameter values conditional on a given model ${\mathscr{M}}_{j}$. When multiple models are in play, we can extend Bayes’ theorem and use the data to update the relative plausibility of each of the candidate models. For the case of two models, ${\mathscr{M}}_{0}$ and ${\mathscr{M}}_{1}$, Equation 4 shows how the prior model odds (i.e., the relative plausibility of ${\mathscr{M}}_{0}$ and ${\mathscr{M}}_{1}$ before seeing the data) are updated to posterior model odds (i.e., the relative plausibility of ${\mathscr{M}}_{0}$ and ${\mathscr{M}}_{1}$ after seeing the data). The change from prior to posterior odds is given by the *Bayes factor* (e.g., Jeffreys 1961; Kass and Raftery 1995), which indicates the models’ relative predictive performance for the data at hand (i.e., the ratio of marginal likelihoods):

4 $$ \begin{array}{@{}rcl@{}} \underbrace{\frac{p(\mathcal{M}_{1} \mid \mathcal{D})}{p(\mathcal{M}_{0} \mid \mathcal{D})}}_{\text{Posterior model odds}} &= \quad \underbrace{\frac{p(\mathcal{M}_{1})}{p(\mathcal{M}_{0})}}_{\text{Prior model odds}} \times \quad \underbrace{\frac{p(\mathcal{D} \mid \mathcal{M}_{1})}{p(\mathcal{D} \mid \mathcal{M}_{0})}}_{\substack{\text{Bayes factor} \\ \text{BF}_{10}}}. \end{array} $$

When the Bayes factor BF_10_ is 4 this indicates that the data are 4 times more likely under ${\mathscr{M}}_{1}$ than ${\mathscr{M}}_{0}$. The Bayes factor subscripts indicate which model is in the numerator and denominator; for instance, if BF_10_ = 0.20, then 1 / BF_10_ = BF_01_ = 5, which means that the data are 5 times more likely under ${\mathscr{M}}_{0}$ than under ${\mathscr{M}}_{1}$ (Jeffreys, 1939). There exist several categorization schemes to quantify the evidence associated with particular ranges of values (e.g., Jeffreys 1961; Kass and Raftery 1995). Table 1 provides one such scheme.

**Table 1:** A scheme for categorizing the strength of a Bayes factor (from Lee and Wagenmakers (2013), based on Jeffreys (1961)). Note that the Bayes factor is a continuous measure of evidence and that the thresholds provided here (and in other schemes) are only meant as a heuristic guide to facilitate interpretation and not as a definite cutoff

Bayes factor BF_10_			Interpretation
	>	100	Extreme evidence for ${\mathscr{M}}_{1}$
30	−	100	Very strong evidence for ${\mathscr{M}}_{1}$
10	−	30	Strong evidence for ${\mathscr{M}}_{1}$
3	−	10	Moderate evidence for ${\mathscr{M}}_{1}$
1	−	3	Anecdotal evidence for ${\mathscr{M}}_{1}$
	1		No evidence
1/3	−	1	Anecdotal evidence for ${\mathscr{M}}_{0}$
1/10	−	1/3	Moderate evidence for ${\mathscr{M}}_{0}$
1/30	−	1/10	Strong evidence for ${\mathscr{M}}_{0}$
1/100	−	1/10	Very strong evidence for ${\mathscr{M}}_{0}$
	<	1/100	Extreme evidence for ${\mathscr{M}}_{0}$

With more than two candidate models in the set, the posterior model probability for model ${\mathscr{M}}_{j}$ is given by

$$ \begin{array}{@{}rcl@{}} p(\mathcal{M}_{j}\mid\mathcal{D}) = \frac{p(\mathcal{D}\mid\mathcal{M}_{j})p(\mathcal{M}_{j})}{{\sum}_{i}p(\mathcal{D}\mid\mathcal{M}_{i})p(\mathcal{M}_{i})}. \end{array} $$

This can also be written as a function of the Bayes factor relative to the null model:

$$ \begin{array}{@{}rcl@{}} p(\mathcal{M}_{j}\mid\mathcal{D}) = \frac{\text{BF}_{j0}\quad(\mathcal{M}_{j})}{{\sum}_{i}\text{BF}_{i0}\quad(\mathcal{M}_{i})}. \end{array} $$

The change from prior to posterior model odds quantifies the evidence $\text {BF}_{{\mathscr{M}}_{j}}$ that the data provide for a particular model *j*. The prior model odds are given by $\nicefrac {p({\mathscr{M}}_{j})}{1 - p({\mathscr{M}}_{j})}$ and the posterior model odds are given by $\nicefrac {p({\mathscr{M}}_{j}\mid \mathcal {D})}{1 - p({\mathscr{M}}_{j}\mid \mathcal {D})}$. The change in odds is obtained by dividing the posterior model odds by the prior model odds:


Bayes factors generally depend on the prior distribution for the parameter values. In contrast to estimation, the data do not overwhelm the prior because the Bayes factor quantifies relative predictive performance of two models on a data set.^1^ This is desirable because complex models usually yield many poor predictions and therefore the Bayes factor inherently penalizes complexity and favors parsimony (Jeffreys, 1961). However, without reliable information suitable for constructing a prior, the relation between Bayes factors and priors introduces the need for default prior distributions.

There are two types of prior distributions that need to be decided upon. The first type of prior distribution is the *model prior*, which assigns a prior probability to each model that is considered. For the time being, we only consider a uniform model prior so that all models are a-priori equally likely. Alternative model priors are discussed in the section *Prior Sensitivity*.

The second type of prior distribution is the prior on parameters. A popular choice of default prior distributions for parameters *β* in linear regression is the Jeffreys–Zellner–Siow (JZS) prior (i.e., a multivariate Cauchy distribution on the beta coefficients)

which is also used in the implementation shown later. The JZS prior fulfills several desiderata (see Arnold Zellner and Siow, 1980; Zellner 1986b; Liang, Paulo, Molina, Clyde, and Berger, 2008) for information on the JZS-prior, see Rouder and Morey (2012) for default priors in Bayesian linear regression, and see Ly, Verhagen, and Wagenmakers, (2016) for a general introduction on default Bayes factor hypothesis tests). An example of such a desideratum is that the Bayes factor is the same regardless of the units of measurement (e.g., the Bayes factor is the same when response time is measured in milliseconds or years; for more information see (Bayarri, Berger, Forte, García-Donato, et al., 2012)). This desideratum is satisfied by assigning a Jeffreys prior to the residual variance *σ*^2^,

that is, *p*(*σ*^2^) is proportional to 1/*σ*^2^.

Other methods included in JASP are the Akaike Information Criterion (AIC; Akaike, 1973), the Bayesian Information Criterion (BIC; Schwarz, 1978), the *g*-prior (Zellner, 1986a), the hyper-*g* prior (Liang et al., 2008), the hyper-*g*-Laplace prior which is the same as the hyper-g prior but uses a Laplace approximation, and the hyper-*g*-*n* prior which uses a hyper-g/n prior (Liang et al., 2008). In addition, two methods are available that use a *g*-prior and automatically choose a value for *g*. Empirical Bayes “global” uses an EM algorithm to find a suitable value for *g* while empirical Bayes “local” uses the maximum likelihood estimate for each individual model as value for *g* (Clyde & George, 2000). We revisit the possible use of these alternative methods when we discuss robustness.

### Bayesian multi-model inference

As before, assume that there are multiple models in play, each with their own set of predictors. In the previous section we have seen that the posterior model probabilities can be obtained by assessing each model’s plausibility and predictive performance, relative to that of the other models in the set. When the results point to a single dominant model, then it is legitimate to consider only that model for inference. When this is not the case, however, inference about the predictors needs to take into account multiple models at the same time. We consider two important questions: (1) what predictors should be included to account for the dependent variable? and (2) what have we learned about the regression coefficients for the predictors? In multi-model inference, these questions can be addressed by summing and averaging across the model space, respectively.

First, consider the question ‘if we want to predict Happiness, do we need the predictor Wealth?’ There may be thousands of regression models, half of which include Wealth as a predictor, and half of which do not. In BMA we can quantify the overall support for the predictor Wealth by summing all posterior model probabilities for the models that include Wealth:

$$ \begin{array}{@{}rcl@{}} p(\text{incl}_{\beta_{j}}\mid \mathcal{D}) = \sum\limits_{\mathcal{M}_{j}: \beta_{j} \in \mathcal{M}_{j}} p(\mathcal{M}_{j} \mid \mathcal{D}) \end{array} $$

If the summed prior probability of models including Wealth is 0.50, and the summed posterior probability is 0.95, then the inclusion Bayes factor is 19. That is:

$$ \begin{array}{@{}rcl@{}} \frac{p(\text{incl}_{\beta_{j}}\mid \mathcal{D})}{p(\text{excl}_{\beta_{j}}\mid \mathcal{D})} = \frac{p(\mathcal{D}\mid\text{incl}_{\beta_{j}})}{p(\mathcal{D}\mid\text{excl}_{\beta_{j}})} \frac{p(\text{incl}_{\beta_{j}})}{p(\text{excl}_{\beta_{j}})} \end{array} $$

Second, consider the question ‘what have we learned about the regression coefficient for the predictor Wealth?’ In the models that do not feature Wealth, this coefficient can be considered zero; in the models that do feature Wealth, the coefficient has a posterior distribution, but a different one for each model. In BMA, we can provide an overall impression of our knowledge about the coefficient by averaging the parameter values across all of the models, using the posterior model probabilities as weights (e.g., Ghosh 2015; Raftery et al. 1997). Intuitively, one can first sample a model (using the posterior model probabilities) and then, from that model, draw a value of the regression coefficient from the posterior distribution for that model; repeating this very many times gives a model-averaged posterior distribution for the regression coefficient of interest. Specifically, we have:

$$ \begin{array}{@{}rcl@{}} p(\beta \mid \mathcal{D}) = \sum\limits_{j}p(\beta \mid \mathcal{D}, \mathcal{M}_{j})  p(\mathcal{M}_{j} \mid \mathcal{D}) \end{array} $$

The same procedure for sampling from the posterior distribution of the regression coefficients can be used to obtain a distribution over model-based predictions. Letting $\hat {y}_{i}$ denote a prediction for outcome *i* we obtain:

$$ \begin{array}{@{}rcl@{}} p(\hat{y}_{i} \mid \mathcal{D}) = \sum\limits_{j}p(\hat{y}_{i} \mid \mathcal{D}, \mathcal{M}_{j})  p(\mathcal{M}_{j} \mid \mathcal{D}) \end{array} $$

Here, one may use the observed values for the predictors to obtain fits for the observed values of the criterion variable, or one can use new values for the predictors to obtain predictions for unseen values of the criterion variable. Note that the predictions and the residuals are random variables endowed with probability distributions, rather than single values.

A complementary method is to base all inference on the *median probability model* (Barbieri, Berger, et al., 2004) which includes all predictors that have posterior inclusion probabilities larger than or equal to 0.5. This method is implemented both in BAS and in JASP.

Although BMA is theoretically straightforward, considerable practical challenges need to be overcome. The main challenge is that the model space can be truly enormous, and consequently even advanced computational methods can grind to a halt. Fortunately, the computational challenge surrounding Bayesian multi-model inference in linear regression has been mostly overcome by a recent method called Bayesian Adaptive Sampling (BAS Clyde, Ghosh, & Littman, 2011). In principle, BAS tries to enumerate the model space if *p* ≤ 20. However, if the model space is too large to enumerate –when *p* > 20 implying that there are more than 1,048,576 models to consider– BAS uses an efficient method for sampling from the model space without replacement. An open-source implementation of BAS is available for R (Core Team (2018); package ‘BAS’, Clyde (2018)) and the methodology is also accessible with a graphical user interface in JASP (JASP Team, 2020).

## Example: World happiness data

To showcase Bayesian multi-model inference for linear regression we consider data from the World Happiness Report of 2018. The data set can be obtained from the appendix of http://worldhappiness.report/ed/2018/. An annotated .jasp file of the analysis detailed below can be found at https://osf.io/5dmj7/. The goal of the analysis is to examine which variables are related to Happiness, and what is the strength of the relation. First we briefly describe the data set.

The World Happiness Data is put together yearly by Gallup, a research-based consulting company. Gallup regularly conducts public opinion polls and annually conducts interviews with a large number of inhabitants of many different countries.^2^ The happiness of the interviewees was assessed with the Cantril Self-Anchoring Striving Scale (Glatzer and Gulyas, 2014). In addition, interviewees were asked about a variety of topics and the obtained data are distilled into six variables that may relate to happiness. A description of these six variables is given in Table 2.

**Table 2:** Description of the predictor variables for the Gallup World Happiness Data. For a more detailed description of the variables see technical box 1 of Gallop’s complete report

Predictor	Abbreviation	Description
GDP per Capita	W	The relative purchasing power of inhabitants of a country, based on data from the World Bank.
Life expectancy	Le	Life expectancy based on data from the World Health Organization.
Social support	Ss	The nation-wide average of responses to the question: ‘If you were in trouble, do you have relatives or friends you can count on to help whenever you need them, or not?’
Freedom	F	The nation-wide average to the question: ‘Are you satisfied or dissatisfied with your freedom to choose what you do with your life?’
Generosity	Ge	The nation-wide average ‘Have you donated to a charity in the last month?’
Perception of corruption	Poc	The nation-wide average to the questions ‘Is corruption widespread throughout the government or not?’ and ‘Is corruption widespread within businesses or not?’.

We first analyze the data using a standard Bayesian multi-model approach, which is then extended to deal with interaction effects, nuisance variables included in all models, and robustness checks.

**Fig. 4:**
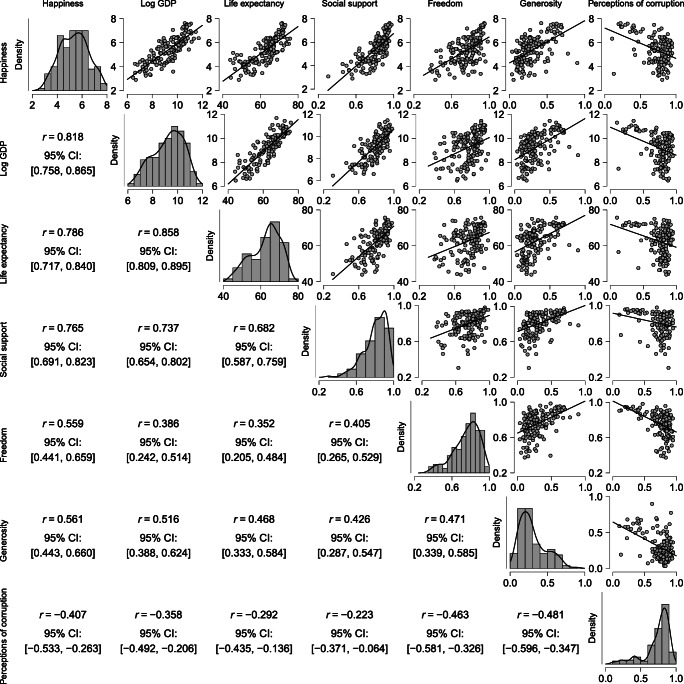
A matrix-plot of all variables in the World Happiness Data. The diagonal plots are the density estimates of the individual variables. The above-diagonal plots are pairwise scatter plots of two variables, where the straight line represent the correlation between them. In the first row, Happiness score (*y*-axes) is plotted against all independent variables (*x*-axes). Below the diagonal the Pearson correlations are displayed. All relations appear approximately linear by eye. Figure from JASP

Before carrying out any analyses it is critical to check the model assumptions. We investigate the assumption of linearity by plotting the entire set of independent variables against the dependent variable, as shown in Fig. 4. To replicate Fig. 4, open JASP and load the data, go to Descriptives, first drag your dependent variable and then all independent variables.^3^ Then under Plots click Correlation plot.

**Fig. 5:**
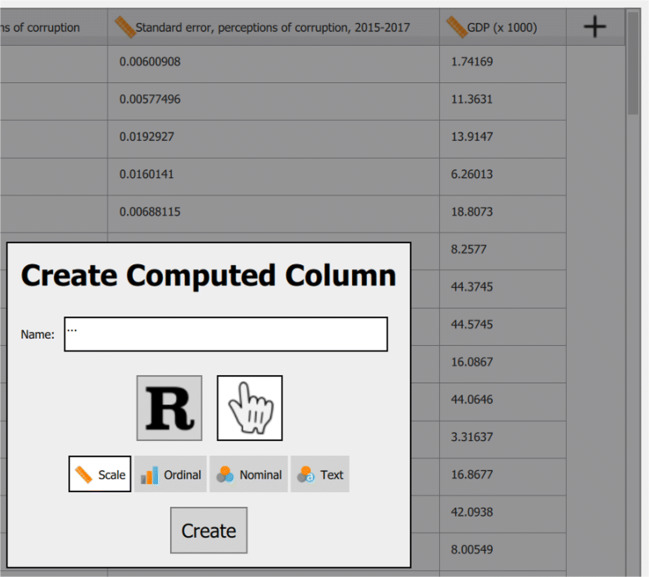
Compute a new column in JASP by clicking on the ‘+’ in the top right of the data view

Figure 4 shows that all relations between the covariates and Happiness are approximately linear. Initially, the relation between Happiness and Wealth was nonlinear (see Fig. 1), but after log-transforming Wealth this assumption no longer appears violated (as shown in Fig. 4). Transforming a variable in JASP can be done by going to the data view, scrolling all the way to the right and selecting Compute Columns. Next, we can create a new variable, either using a drag and drop scheme or using R-code. This is shown in Fig. 5.

**Fig. 6:**
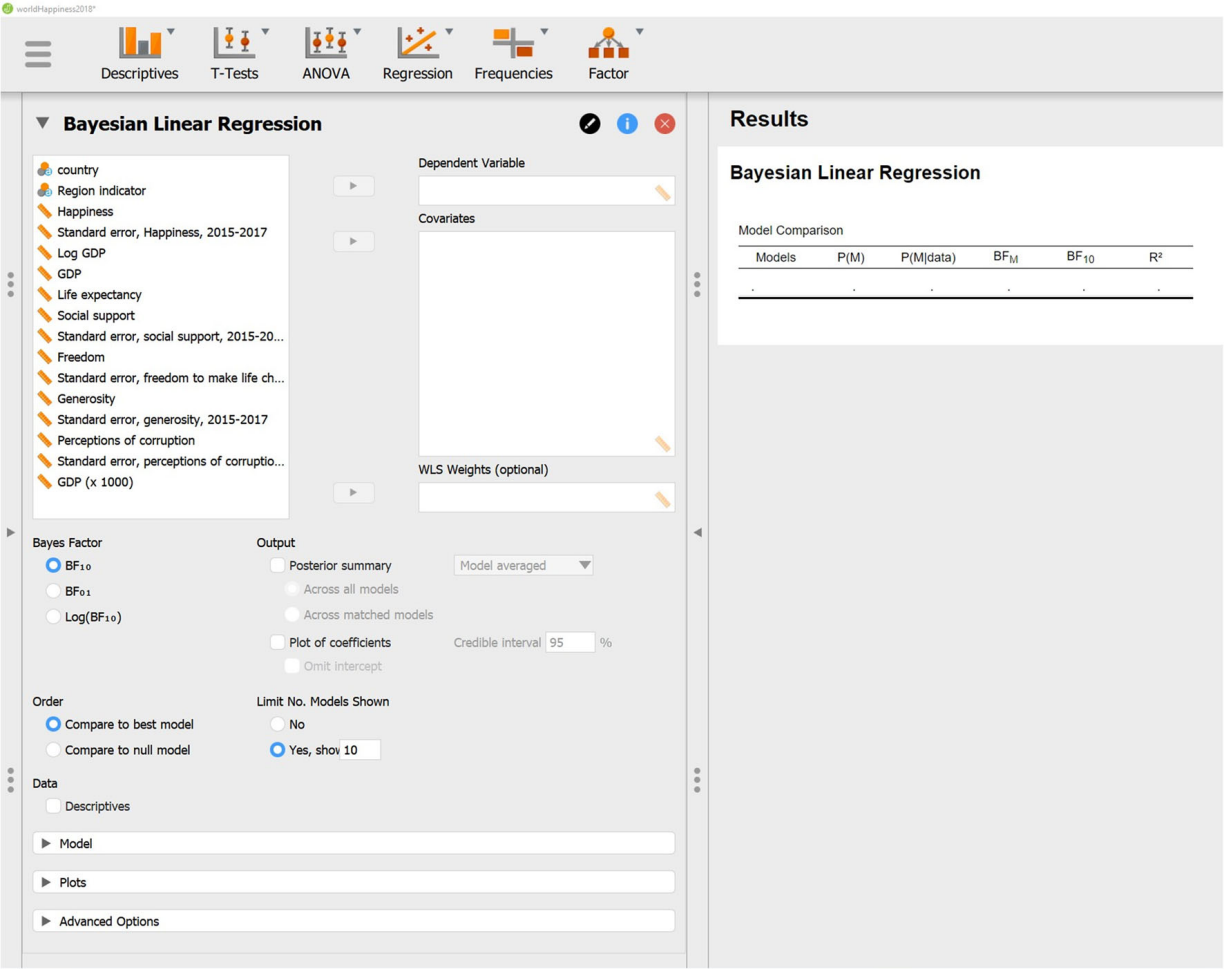
Screenshot of Bayesian linear regression in JASP. The left panel shows the input fields; once these are populated, output will appear in the panel on the right

The other key assumption –normally distributed residuals– can only be studied after executing the analysis. To execute the analysis in JASP, we go to the Regression menu and click on Bayesian Linear Regression. Fig. 6 shows the resulting interface.

**Fig. 7:**
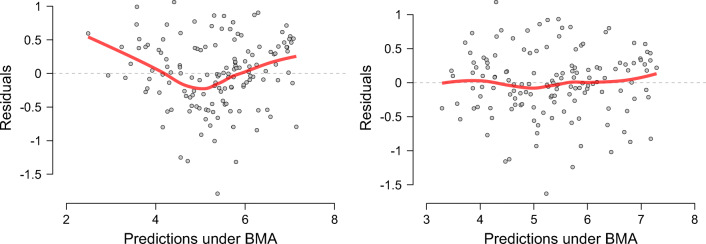
Residuals vs Predictions for the World Happiness data set for the model without (left panel) and with (right panel) the interaction effect of Life expectancy and Social support. The red line is a smoothed estimate of the mean at each point and is ideally completely flat. Figures from JASP

We enter the data by dragging Happiness to the box labeled Dependent Variable and by dragging the independent variables to the box labeled Covariates. As soon as the data are entered the analysis is carried out and the table on the right of Fig. 6 is filled out. Before interpreting the results we assess whether the residuals are approximately normally distributed. To do so, we go to Plots and check Residuals vs. fitted. This produces the left panel of Fig. 7, which shows there is still structure in the residuals that is not captured by the model. We included a two-way interactions between Life expectancy and Social support.^4^ This is motivated by the following comment in Gallop’s report (page 21):

“*There are also likely to be vicious or virtuous circles, with two-way linkages among the variables. For example, there is much evidence that those who have happier lives are likely to live longer, be more trusting, be more cooperative, and be generally better able to meet life’s demands. This will feed back to improve health, GDP, generosity, corruption, and sense of freedom.*” (original in italics)

After confirming that the assumptions of linear regression have been met, we can investigate the results. No further action is required; as soon as the data were entered, JASP executed the analysis and displayed the results in an output table. The results for the ten models with the highest posterior probability are shown in Table 3.

**Table 3:** The 10 best models from the Bayesian linear regression for the Gallup World Happiness Data

Models	$P\left ({\mathscr{M}}\right )$	$P\left ({\mathscr{M}}\mid \mathcal {D} \right )$	BF$_{{\mathscr{M}}}$	BF_01_	*R*^2^
W + Le + Ss + F + Le * Ss	0.013	0.759	248.244	1.000	0.821
W + Le + Ss + F + Ge + Le * Ss	0.013	0.097	8.531	7.783	0.822
W + Le + Ss + F + Poc + Le * Ss	0.013	0.093	8.101	8.157	0.822
Le + Ss + F + Le * Ss	0.013	0.027	2.233	27.591	0.805
W + Le + Ss + F + Ge + Poc + Le * Ss	0.013	0.012	0.924	65.617	0.823
Le + Ss + F + Ge + Le * Ss	0.013	0.005	0.413	145.922	0.807
Le + Ss + F + Poc + Le * Ss	0.013	0.004	0.329	182.965	0.807
W + Le + Ss + F	0.013	6.961*e* − 4	0.055	1089.774	0.794
Le + Ss + F + Ge + Poc + Le * Ss	0.013	6.672*e* − 4	0.053	1137.027	0.808
W + Le + Ss + F + Poc	0.013	3.179*e* − 4	0.025	2386.195	0.799

The leftmost column shows the model specification, where each variable is abbreviated as in Table 2. The second column gives the prior model probabilities; the third the posterior model probabilities; the fourth the change from prior to posterior model odds; the fifth the Bayes factor of the best model over the model in that row; and the last the *R*^2^, the explained variance of each model. Results for all 80 models are presented in the appendix, Table 9

Table 3 shows that the ten best models all contain Life expectancy, Social support, and Freedom, which suggests that these predictors are important to account for Happiness. Also, note that the Bayes factor BF_01_, which quantifies a model’s relative predictive performance, does not always prefer models with higher explained variance *R*^2^, which quantifies a model’s goodness-of-fit. For instance, *R*^2^ is necessarily highest for the full model that contains all seven predictors (row 5 in Table 3); however, the Bayes factor indicates that the predictive performance of this relatively complex model is about 66 times worse than that of the model that contains only Wealth, Life Expectancy, Social support, Freedom, and the interaction between Life expectancy and Social support.

With many different models it can be challenging to quantify the relevance of individual predictors by showing all models as in Table 3 (and its complete version with all 80 models). In model-averaging, the solution is to take into account all models simultaneously. This can be accomplished in JASP by ticking Posterior summary in the input panel and selecting the option Model averaged. The output, shown here in Table 4, provides a summary of the predictor inclusion probabilities and the posterior distributions averaged across all models.

**Table 4:** Model-averaged posterior summary for linear regression coefficients of the Gallup World Happiness Data

						95% CI	
Coefficient	Mean	SD	$P\left (\text {incl}\right )$	$P\left (\text {incl}|\mathcal {D} \right )$	BF_incl_	Lower	Upper
Intercept	5.346	0.041	1.000	1.000	1.000	5.265	5.421
W	0.263	0.094	0.500	0.962	25.616	0.000	0.393
Le	− 0.110	0.035	0.600	1.000	2875	− 0.183	− 0.050
Ss	− 8.545	2.556	0.600	1.000	131213	− 13.688	− 4.167
F	1.699	0.345	0.500	1.000	3772	1.067	2.327
Ge	0.028	0.127	0.500	0.115	0.130	− 0.037	0.390
Poc	− 0.022	0.112	0.500	0.110	0.124	− 0.306	0.043
Le * Ss	0.189	0.044	0.200	0.998	2475	0.105	0.267

The leftmost column denotes the predictor (abbreviations are shown in Table 2). The columns ‘mean’ and ‘sd’ represent the respective posterior mean and standard deviation of the parameter after model averaging. $P\left (\text {incl}\right )$ denotes the prior inclusion probability and $P\left (\text {incl}\mid \text {data}\right )$ denotes the posterior inclusion probability. The change from prior to posterior inclusion odds is given by the inclusion Bayes factor $\left (\text {BF}_{\text {incl}}\right )$. The last two columns represent a 95% central credible interval (CI) for the parameters

Table 4 confirms our initial impression about the importance of Wealth, Life expectancy, Social Support, Freedom, and the interaction between Life expectancy and Social Support. Each of these predictors are relevant for predicting Happiness, as indicated by the fact that the posterior inclusion probabilities (0.962, 1.000, 1.000, 1.000, and 0.998 respectively) are all near 1.^5^ On the other hand, there is evidence against the relevance of Generosity and Perception of Corruption: the data lowered the inclusion probabilities from 0.5 to about 0.1. The median probability model (i.e., the model that includes all predictors with a posterior inclusion probability larger than 0.5, Barbieri et al., (2004)) consists of Wealth, Life expectancy, Social support, Freedom, and the interaction between Life expectancy and Social support. To obtain the posterior summary for the median probability model, click on the menu that says Model averaged and change it to Median model.

Note that the prior inclusion probabilities are not equal for all coefficients. This happens because JASP automatically excludes models with interactions effects but without their corresponding main effects, as dictated by the principle of marginality (for details see Nelder (1977)).

Thus the prior inclusion probability, $P\left (\text {incl}\right )$ is still obtained by adding up the prior probability of all models that contain a particular coefficient, but for interaction effects there are simply fewer models that are added up. This is further explained in the section *Including Interaction Effects*.

**Fig. 8:**
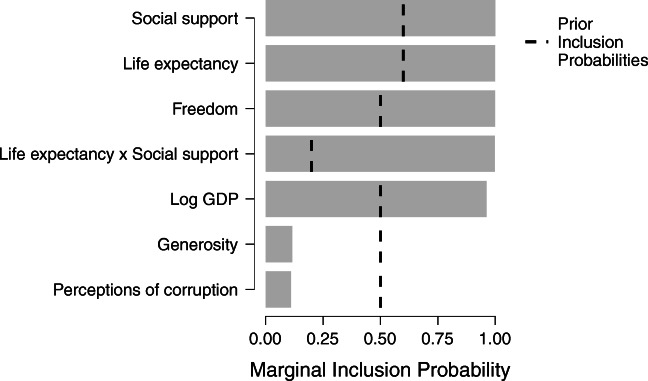
Bar graph of posterior inclusion probabilities for the Bayesian linear regression of the Gallup World Happiness Data. The dashed line represents the prior inclusion probabilities. Figure from JASP

The change from prior to posterior inclusion probabilities can be visualized by selecting Plots and ticking Inclusion probabilities, which produces the bar graph shown in Fig. 8.

**Fig. 9:**
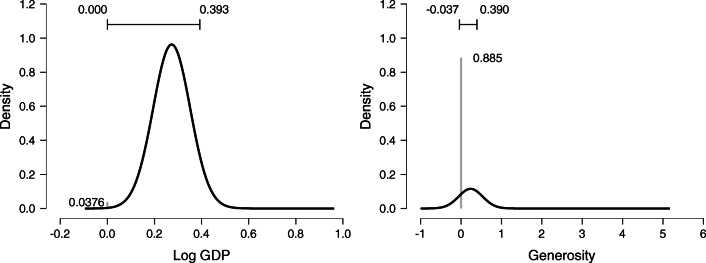
The model-averaged posterior of Wealth expressed in GDP (left) and Generosity (right). In the left panel, the number in the bottom left represents the posterior exclusion probability. In the right panel, the posterior exclusion probability is much larger. In both panels, the horizontal bar on top represents the 95% central credible interval. Figures from JASP

In addition to providing the inclusion probabilities, Table 4 also summarizes the model-averaged posterior distributions using four statistics (i.e., mean, sd, and the lower and upper values of an x% central credible interval). The complete model-averaged posteriors can be visualized by selecting Plots and ticking Marginal posterior distributions. For example, the posterior distribution for the regression coefficient of Wealth is shown in the left panel of Fig. 9. The right panel of Fig. 9 shows the model-averaged posterior for the regression coefficient of Generosity; the spike at zero corresponds to the absence of an effect, and its height reflects the predictor’s posterior exclusion probability. The horizontal bar above the distribution shows the 95% central credible interval.

To summarize, the Bayesian model-averaged analysis showed that the most important predictors in the Gallup World Happiness Data are Wealth, Social Support, Life expectancy, and Freedom. There is weak evidence that Generosity and Perception of Corruption are not relevant for predicting Happiness.

### Including interaction effects

**Fig. 10:**
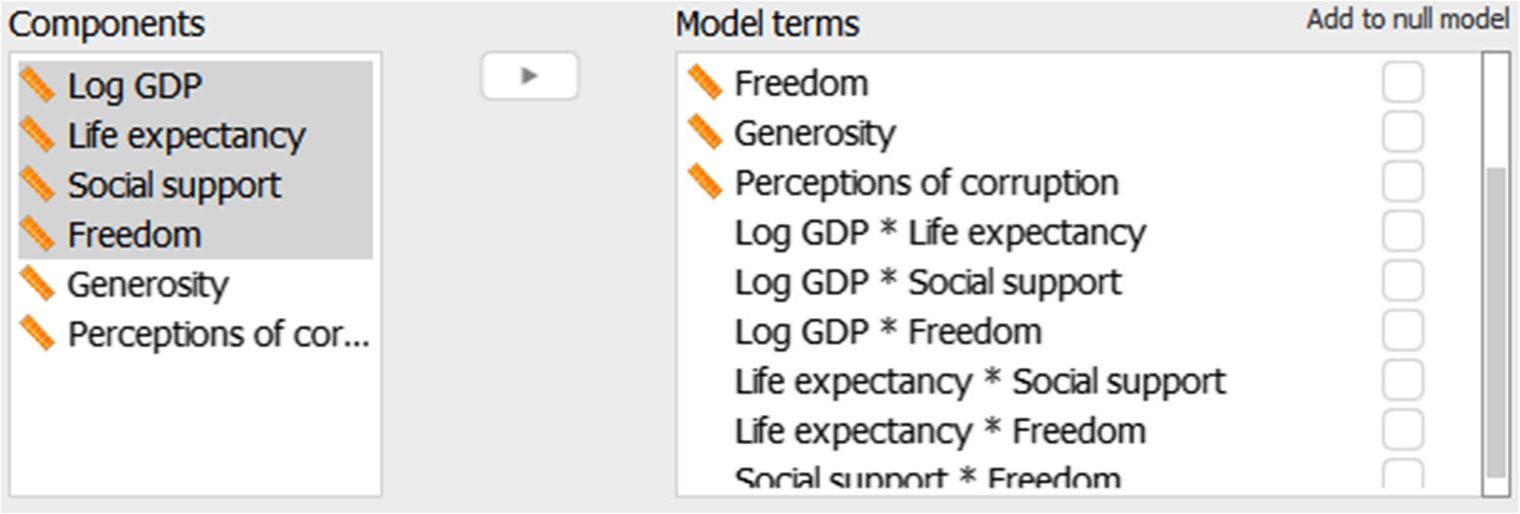
Model component view. By selecting multiple variables in the left panel and dragging these to the right panel, all interactions between the selected variables are included in the model. By ticking the box ‘Add to null model’ the associated variable is included in all models

In regression analysis we are often not interested solely in the main effects of the predictors, but also in the interaction effects. For instance, suppose that for the analysis of the Gallup World Happiness Data we wish to consider the two-way interactions between Wealth, Social Support, Freedom, and Life Expectancy. To do this we click on Model and select all variables of interest under Components (use ctrl /  or Shift to select multiple variables) and drag them to Model terms. JASP then automatically includes all possible interactions between the selected variables in the Model terms on the right. To exclude higher order interactions, we select these in Model terms and click the arrow or drag them to Components. The result is shown in Fig. 10.

As soon as the interaction effects are added to the model, JASP updates the output.^6^ Since the interaction effects account for 6 new predictors there are now 12 predictors in total and 468 models to consider. There are not 2^12^ = 4096 models, because JASP automatically excludes models with interactions effects but without their corresponding main effects, as dictated by the principle of marginality (Nelder, 1977). The updated posterior summary is shown in Table 5.

**Table 5:** Model-averaged posterior summary for linear regression coefficients of the Gallup World Happiness Data, including two-way interaction effects between Wealth, Social Support, Freedom, and Life Expectancy

						95% CI	
Coefficient	Mean	SD	$P\left (\text {incl}\right )$	$P\left (\text {incl}|\mathcal {D}\right )$	BF_incl_	Lower	Upper
Intercept	5.346	0.041	1.000	1.000	1.000	5.260	5.425
W	0.233	0.599	0.841	0.982	10.490	− 0.945	1.753
Le	− 0.122	0.084	0.841	0.997	54.237	− 0.288	0.051
Ss	− 6.576	4.190	0.841	1.000	3057.789	− 12.821	3.223
F	− 0.469	2.901	0.841	1.000	1695.479	− 6.258	2.608
Ge	0.021	0.117	0.500	0.110	0.124	− 0.136	0.236
Poc	− 0.015	0.108	0.500	0.106	0.119	− 0.409	0.058
W * Le	0.002	0.006	0.363	0.200	0.438	− 0.0002	0.019
W * Ss	− 0.186	0.599	0.363	0.241	0.557	− 1.969	0.660
W * F	0.076	0.237	0.363	0.181	0.389	− 0.066	0.788
Le * Ss	0.168	0.116	0.363	0.831	8.612	0.000	0.402
Le * F	0.011	0.035	0.363	0.180	0.385	− 0.0001	0.117
Ss * F	1.072	2.562	0.363	0.228	0.517	− 0.263	8.086

Table 5 shows that Wealth, Social Support, Life expectancy, and Freedom are important for predicting Happiness, as indicated by the posterior inclusions probabilities. For almost all interaction effects, the posterior inclusion probabilities are smaller than the prior inclusion probabilities, indicating that the data provide evidence against these effects. The interaction effect between Life Expectancy and Social Support somewhat improves the model (BF_incl_ = 8.612).

Comparing the main effects in Table 4 to those in Table 5, it might appear surprising that the support for including the predictors decreased for all variables. For example, the inclusion Bayes factor for Life Expectancy decreased from about 2875 to 54, Wealth decreased from about 26 to 10, and the interaction between Life Expectancy and Social support decreased from about 2475 to 9. The cause for these change lies in the added interaction effects. All interaction effects with Wealth led to poorly performing models, as illustrated by the low inclusion Bayes factors for all interaction effects with Wealth. As a consequence, the inclusion Bayes factor for Wealth also suffered, since 312 out of the 396 models considered to calculate the inclusion Bayes factor contained interaction effects with Wealth.

The effect of model averaging on parameter estimation is clearly present when comparing the 95% credible intervals in Tables 4 and 5. For instance, the credible interval for Freedom was [1.06,2.35] in Table 4 but widens to [− 6.3,2.6] in Table 5. There are two reasons for this increase in uncertainty. First, the posterior probability of the best model is only 0.223, compared to 0.759 in Table 3 (see the online supplement for all posterior model probabilities). This means that other models contribute substantially to the model-averaged posterior, which increases the uncertainty in the parameter estimates. Second, the results in Table 5 are based on a larger model space, which potentially leads to a wider range of possible estimates and hence increases the associated uncertainty.

The instability of the results due to changing the model space is no reason for concern; rather, it demonstrates the importance of considering all models and dealing with model uncertainty appropriately. The example above does show, however, that some rationale should be provided for the model space. Here, we did not properly motivate the inclusion of the interaction effects because we wanted to demonstrate the effect of model uncertainty on the results. Instead, one should decide upon the the model space before executing the analysis and ideally preregister the model space on the basis of substantive considerations.

### Including nuisance predictors in all models

Another common procedure in the toolkit of linear regression is to include a number of nuisance predictors in all models in management sience this is sometimes called hierarchical regression; see also Petrocelli (2003) and Andraszewicz et al., (2015). Subsequently, the goal is to assess the contribution of the predictor(s) of interest over and above the contribution from the nuisance predictors. For example, we could have included Wealth in all models, for instance because we already know that Wealth has a large effect, but we are not interested in that effect – we are interested in what the other predictors add on top of Wealth. To add Wealth as a nuisance variable to the model, we go to Model and check the box under Add to null model for Wealth (see Fig. 10). As with interaction effects, JASP updates the results immediately and produces a model comparison table similar to Table 3. Note that the Bayes factor BF_01_ in the fifth column of Table 3 by default compares all models to the *best* model. When including nuisance predictors, we are more interested in how much the models improve compared to the null model. We can change the default setting by going to Order and selecting Compare to null model. This changes the Bayes factor column such that all models are compared to the null model instead of to the best model. The resulting table is shown in Table 6. Since we now compare all models to the null model, the null model is always shown in the first row.

**Table 6:** The 10 best models from the Bayesian linear regression for the Gallup World Happiness Data, where the nuisance predictor Wealth is included in all models. The interpretation of the columns is identical to that of Table 3, except that the Bayes factor BF_01_ in the fifth column compares all models to the null model. The table footnote shows a reminder from JASP which variables are specified as nuisance

Models	$P\left ({\mathscr{M}}\right )$	$P\left ({\mathscr{M}}\mid \text {data}\right )$	BF$_{{\mathscr{M}}}$	BF_01_	*R*^2^
Null model (incl. W)	0.031	6.143*e* − 11	1.904*e* − 9	1.000	0.679
Le + Ss + F	0.031	0.439	24.228	7.141*e* + 9	0.794
Le + Ss + F + Poc	0.031	0.200	7.767	3.261*e* + 9	0.799
Le + Ss + F + Ge	0.031	0.169	6.290	2.746*e* + 9	0.799
Ss + F	0.031	0.077	2.572	1.247*e* + 9	0.781
Le + Ss + F + Ge + Poc	0.031	0.043	1.380	6.938*e* + 8	0.802
Ss + F + Poc	0.031	0.032	1.034	5.254*e* + 8	0.786
Ss + F + Ge	0.031	0.030	0.955	4.867*e* + 8	0.786
Ss + F + Ge + Poc	0.031	0.007	0.217	1.131*e* + 8	0.789
Le + F	0.031	0.002	0.057	2.966*e* + 7	0.769

*Note.* All models include Wealth (W)

### Prior sensitivity

#### Priors on parameters

In the previous analyses we used the default JZS prior on the values of the regression coefficients. However, it is generally recommended to investigate the robustness of the results against the choice of prior (van Doorn et al., 2019). To investigate robustness, one typically uses the same family of distributions but varies the prior width. A wider prior will imply more spread-out a-priori uncertainty about the effect, whereas a more narrow prior implies that the a-priori belief about the effect is more concentrated near zero. To adjust the prior, we go to Advanced options and under Prior change the value after JZS. This value is generally referred to as the scale of the JZS prior. The default choice in JASP is a JZS with a scale of 1/8. This corresponds to the default choice used in other software, for example the R package “BayesFactor” (Morey and Rouder, 2018). If the JZS scale in JASP is *s*, the corresponding scale for the “BayesFactor” package is $\sqrt {2s}$.

**Fig. 11:**
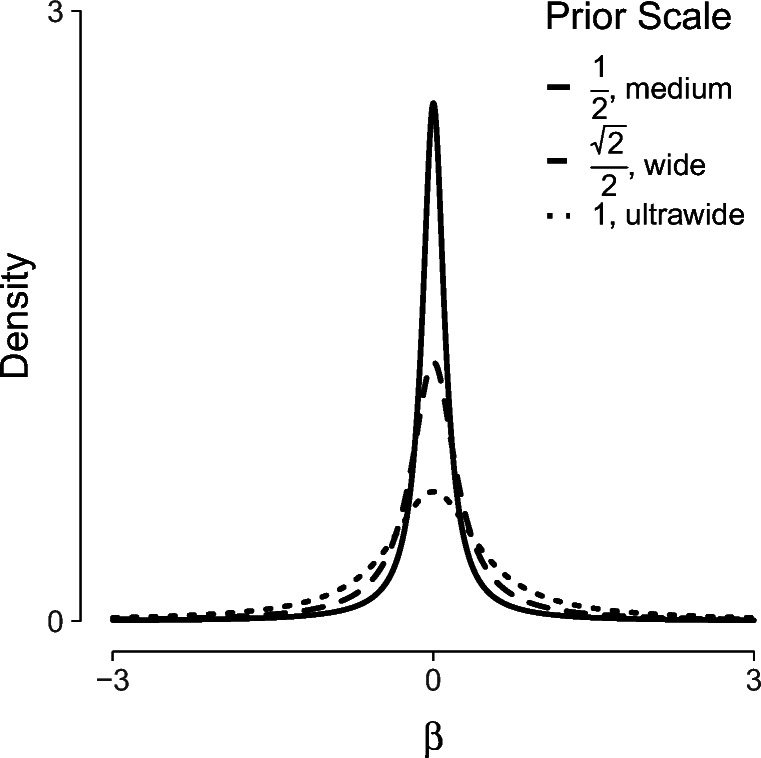
Marginal prior distribution on the regression coefficients (*β*). The different line types represent different scales for the prior. As the scale increases the probability mass near zero decreases and the mass on more extreme values increases

Commonly used values for the larger scales are 1/4 and 1/2, respectively referred to as “wide” and “ultrawide” priors (Wagenmakers et al., 2018; Morey & Rouder, 2018). Figure 11 shows the marginal prior distribution for the regression coefficients *β* for these three scales. Under Advanced options it is also possible to select other prior distributions than the JZS. However, we recommend against doing so without proper motivation (see e.g., Consonni, Fouskakis, Liseo, Ntzoufras, et al., 2018; Liang et al., 2008; Bayarri et al., 2012).

We repeated the main analysis with a JZS scale of 1/4 and 1/2 but the posterior inclusion probabilities, see Table 7, did not change in a meaningful way (see https://osf.io/5dmj7/ for an annotated .jasp file with the results).

**Table 7:** Posterior inclusion probabilities given different values for the scale of the JZS prior. The intercept is omitted from the comparison as it is included in all models and therefore its inclusion probability is always 1

		$P\left (\text {incl}|\mathcal {D}\right )$		
Coefficient	P(incl)	s = medium	s = wide	s = ultrawide
Log GDP	0.5	0.962	0.962	0.962
Le	0.6	1.000	1.000	1.000
Ss	0.6	1.000	1.000	1.000
F	0.5	1.000	1.000	1.000
G	0.5	0.115	0.114	0.111
Poc	0.5	0.110	0.109	0.106
Le * Ss	0.2	0.998	0.998	0.998

#### Priors on the model space

Aside from adjusting the priors on the coefficients, it is also possible to adjust the prior over the models. An intuitive choice is a uniform model prior, where each model is assigned prior mass equal to one over the number of models considered. This prior was also used in the analyses above. However, if we use a uniform model prior and then compute the prior probability for a model that includes *x* predictors, where *x* goes from 0 to *p*, we do not obtain a uniform prior. Instead, the implied prior over the number of included predictors is bell-shaped with the most mass on models with *p*/2 predictors. Thus, a-priori our prior is biased against sparse models and dense models, and favors something in between.

**Fig. 12:**
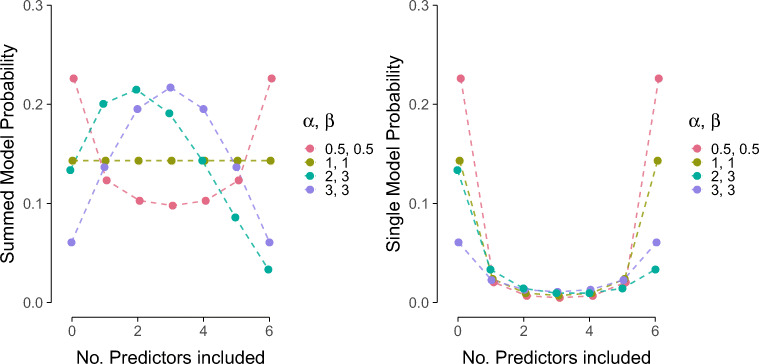
A beta-binomial model prior for a model space with 6 predictors. The left panel shows the beta-binomial distribution where the number of predictors in the model (*x*-axis) is visualized against the total probability of all models with that number of predictors (*y*-axis). The right panel shows how the number of predictors in the model (*x*-axis) influences the prior probability of a single model (*y*-axis). The right panel is obtained by dividing each probability in the left panel by the number of models with that many predictors. The number of models that contain *j* predictors is obtained by calculating $\binom {6}{j}$. This yields for 0 through 6: 1, 6, 15, 20, 15, 6, and 1

**Fig. 13:**
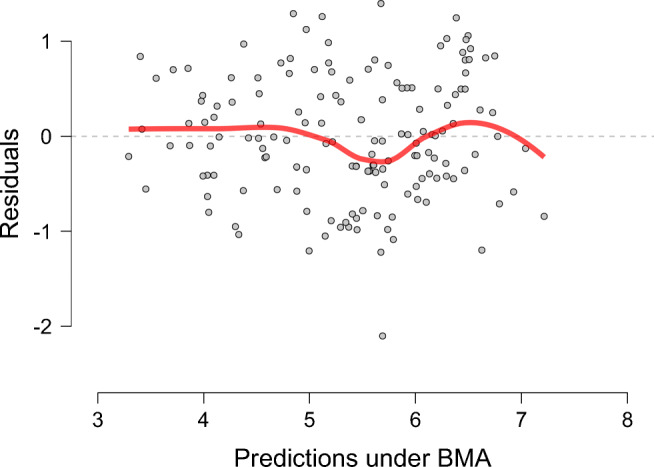
Assumptions checks for Happiness predicted by log-transformed Wealth. In contrast to the right panel of Fig. 2, the red line is completely flat and the variance is approximately constant across the predicted values

A solution to this problem is to use a prior that is uniform over the number of included predictors. This can be achieved by dividing the total probability, 1, into *p* + 1 chunks. The first chunk represents the combined probability of all models that include no predictors, the second chunk represents the combined probability of all models that include one predictor, etc. This model prior commonly referred to as a beta-binomial model prior and can be tweaked using two parameters, *α* and *β*. The left panel of Fig. 12 shows how the total probability is divided for different values of *α* and *β*. The default values in JASP are *α* = *β* = 1.^7^ In the next step, all models within a chunk (i.e. all models with the same number of predictors) are treated as equally likely and the probability of the chunk is distributed uniformly among them. This implies the prior probability of a chunk is divided by the number of models in that chunk. The right panel of Fig. 12 shows the prior model probability for different values of *α* and *β*.

We repeated the main analysis with a Beta-binomial prior. Table 8 shows the inclusion probabilities for an uniform model prior and a beta-binomial model prior. Although the numbers differ, the results are unchanged: The evidence for the inclusion and exclusion of predictors in the model point in the same direction for both priors on the model space. For example, the inclusion Bayes factors that were larger than 1 for a uniform prior on the model space were also larger than 1 for the beta-binomial prior.

**Table 8:** Prior inclusion probabilities, posterior inclusion probabilities, and inclusion Bayes factors for a uniform model prior and a beta-binomial model prior. The intercept is omitted from the comparison as it is included in all models and therefore its inclusion probability is always 1

	Uniform			Beta-binomial		
Coefficient	$P\left (\text {incl}\right )$	$P\left (\text {incl}|\mathcal {D}\right )$	BF_incl_	$P\left (\text {incl}\right )$	$P\left (\text {incl}|\mathcal {D}\right )$	BF_incl_
Log GDP	0.5	0.962	25.616	0.489	0.983	59.024
Le	0.6	1.000	2875	0.556	1.000	8924
Ss	0.6	1.000	131213	0.556	1.000	398502
F	0.5	1.000	3772	0.489	1.000	5775
G	0.5	0.115	0.130	0.489	0.339	0.536
Poc	0.5	0.110	0.124	0.489	0.330	0.515
Le * Ss	0.2	0.998	2475	0.333	0.999	2336

Although much attention goes to the choice of prior distribution, the likelihood of the statistical model is often more important. As stated by Gelman and Robert (2013):

“*It is perhaps merely an accident of history that skeptics and subjectivists alike strain on the gnat of the prior distribution while swallowing the camel that is the likelihood.* ” (italics in original)

In other words, choices about which predictors and interaction effects to consider, choices that influence the likelihood, are more important than the choice of prior distribution. This again stresses the importance to demarcate the model space.

## Discussion

This paper provided a tutorial on Bayesian multi-model inference and aimed to bridge the gap between statistical theory and the applied researcher. Multi-model inference and regression analyses are subject to a number of limitations, which are discussed below.

### Limitations

At the moment of writing, the linear regression procedures as implemented in JASP and BAS do not account for missing values; therefore, missing values are deleted list-wise (i.e., cases with missing values for one or more predictors are omitted from the analysis entirely). However, Bayesian analyses can handle missing values by perceiving them as unknown parameters of the model. That way, the observed value can still contribute to the model and the uncertainty around the missing values is dealt with accordingly (Little & Rubin, 2002, Ch 10).

A general challenge for regression models arises when the predictors are multicollinear, that is, very highly correlated. To illustrate, consider the data of 13 American football punters (Faraway, 2005, available from). The goal is to relate various physical characteristics of the football players to their average punting distance. Relevant predictors are right leg strength, left leg strength, right hamstring flexibility, and left hamstring flexibility. Unsurprisingly, the correlation between the right and left leg predictors is very high. Consequently, models that contain predictors from one leg benefit little when the predictor from the other leg is added on top. Thus, models with predictors for both legs perform poorly compared to models containing information of only one leg. After calculating the inclusion Bayes factors it is unclear whether any specific predictor should be included. Paradoxically, when directly comparing the models, the null model is one of the worst models; it performs about 31.8 times worse than the best model with right hamstring flexibility as the only predictor. See punting.jasp at https://osf.io/5dmj7/ for an annotated analysis. Nonetheless, these results make sense. The model averaged results are unable to distinguish between the correlated predictors because individually they improve the model but jointly they worsen it. For example, the second best model contains right leg strength as a predictor, the fifth best model contains left leg strength as a predictor, but the model that contains both right and left leg strength as predictors ranks 11^th^ out of 16. Hence, there is a lingering uncertainty about which predictor to include, even though directly comparing the different models shows that a model including at least one predictor already performs better than the null model.

Recognizing multicollinearity is always important in linear regression. This does not require much additional work; when creating Fig. 4, the pairwise correlations can also be examined. Another way to assess multicollinearity is by calculating the variance inflation factor (Sheather, 2009, Ch. 6.4).

### Violation of assumptions

If the assumption of linearity appears violated for one or more predictors, some transformations can be used (e.g., a log-transformation). Alternatively, one could try including the square (or cube) of a predictor, and including that in the regression equation to capture any nonlinear relations. This is also known as polynomial regression and can be used to relax the linearity assumption. In JASP, polynomial regression or other transformations can be managed easily using Compute Columns. If the relation between the criterion variable and predictors is innately non-linear, for instance because the criterion variable is binary, generalized linear models can be used. The R package BAS can also be used for multi-model inference for generalized linear models.

If the residuals appear non-normal or heteroscedastic, then there is no clear way how to proceed. Ideally, one first identifies the cause of the violation. Violations can be caused by a single predictor with a nonlinear relation causing misfit, or by multiple predictors. Nonlinearities can be dealt with using the suggestions in the previous paragraph. If the source remains unclear, or is innate to the data, alternative methods can be used. One alternative is to use a probabilistic programming language suited for general Bayesian inference, such as JAGS (Plummer, 2003), NIMBLE (de Valpine et al., 2017), OpenBUGS (Lunn, Spiegelhalter, Thomas, & Best, 2009), or MultiBUGS (Goudie, Turner, De Angelis, & Thomas, 2017), all of which are conceptual descendants of WinBUGS (Lunn, Thomas, Best, & Spiegelhalter, 2000; Ntzoufras, 2009).

The main advantage of probabilistic programming languages is their flexibility: for instance, models can be adjusted to accommodate heteroscedastic residuals (e.g., Reich & Ghosh, 2019, Ch. 4.5.2). These languages also come with disadvantages. First, it is easier to make a mistake – either a programming error, a statistical error, or both. Second, the languages are generic, and because they are not tailored to specific applications they may be relatively inefficient compared to a problem-specific method.

In sum, the goal of this tutorial was to familiarize applied researchers with the theory and practice of Bayesian multi-model inference. By accounting for model uncertainty in regression it is possible to prevent the overconfidence that inevitable arises when all inference is based on a single model. We hope that tutorial will enable applied researchers to use Bayesian multi-model inference in their own work.

## Appendix A: Appendix A: Complete model comparison table

**Table 9:** Bayesian multi-model inference for the World Happiness example: all 80 models. The leftmost column gives the model specification; the second column gives the prior model probabilities; the third the posterior model probabilities; the fourth the change from prior odds to posterior odds; the fifth the Bayes factor relative to the best model; and the last gives R^2^

Models	$P\left ({\mathscr{M}}\right )$	$P\left ({\mathscr{M}}\mid \text {data}\right )$	BF$_{{\mathscr{M}}}$	BF_10_	*R*^2^
W + Le + Ss + F + Le * Ss	0.013	0.759	248.244	1.000	0.821
W + Le + Ss + F + Ge + Le * Ss	0.013	0.097	8.531	7.783	0.822
W + Le + Ss + F + Poc + Le * Ss	0.013	0.093	8.101	8.157	0.822
Le + Ss + F + Le * Ss	0.013	0.027	2.233	27.591	0.805
W + Le + Ss + F + Ge + Poc + Le * Ss	0.013	0.012	0.924	65.617	0.823
Le + Ss + F + Ge + Le * Ss	0.013	0.005	0.413	145.922	0.807
Le + Ss + F + Poc + Le * Ss	0.013	0.004	0.329	182.965	0.807
W + Le + Ss + F	0.013	6.961*e* − 4	0.055	1089.774	0.794
Le + Ss + F + Ge + Poc + Le * Ss	0.013	6.672*e* − 4	0.053	1137.027	0.808
W + Le + Ss + F + Poc	0.013	3.179*e* − 4	0.025	2386.195	0.799
W + Le + Ss + F + Ge	0.013	2.676*e* − 4	0.021	2834.341	0.799
W + Ss + F	0.013	1.216*e* − 4	0.010	6239.227	0.781
W + Le + Ss + Poc + Le * Ss	0.013	8.133*e* − 5	0.006	9327.093	0.795
W + Le + Ss + F + Ge + Poc	0.013	6.763*e* − 5	0.005	11216.690	0.802
W + Le + Ss + Ge + Le * Ss	0.013	6.430*e* − 5	0.005	11796.826	0.794
W + Ss + F + Poc	0.013	5.121*e* − 5	0.004	14813.739	0.786
W + Le + Ss + Le * Ss	0.013	4.945*e* − 5	0.004	15340.968	0.786
W + Ss + F + Ge	0.013	4.745*e* − 5	0.004	15988.688	0.786
W + Le + Ss + Ge + Poc + Le * Ss	0.013	2.911*e* − 5	0.002	26057.578	0.799
Le + Ss + Ge + Le * Ss	0.013	1.404*e* − 5	0.001	54049.136	0.782
Le + Ss + Poc + Le * Ss	0.013	1.313*e* − 5	0.001	57757.710	0.782
W + Ss + F + Ge + Poc	0.013	1.102*e* − 5	8.710*e* − 4	68808.309	0.789
Le + Ss + F	0.013	8.251*e* − 6	6.518*e* − 4	91942.898	0.772
Le + Ss + F + Ge	0.013	8.136*e* − 6	6.427*e* − 4	93244.135	0.780
Le + Ss + F + Poc	0.013	7.467*e* − 6	5.899*e* − 4	101586.552	0.780
Le + Ss + Ge + Poc + Le * Ss	0.013	6.790*e* − 6	5.364*e* − 4	111729.632	0.787
Le + Ss + Le * Ss	0.013	5.554*e* − 6	4.388*e* − 4	136585.291	0.771
W + Le + F	0.013	2.891*e* − 6	2.284*e* − 4	262420.104	0.769
Le + Ss + F + Ge + Poc	0.013	2.704*e* − 6	2.136*e* − 4	280537.628	0.784
W + Le + F + Ge	0.013	9.872*e* − 7	7.799*e* − 5	768432.339	0.773
W + Le + F + Poc	0.013	6.255*e* − 7	4.941*e* − 5	1.213*e* + 6	0.772
W + Le + Ss + Poc	0.013	4.229*e* − 7	3.341*e* − 5	1.794*e* + 6	0.770
W + Le + Ss + Ge + Poc	0.013	4.004*e* − 7	3.163*e* − 5	1.894*e* + 6	0.778
W + F	0.013	2.744*e* − 7	2.168*e* − 5	2.764*e* + 6	0.751
W + Le + Ss + Ge	0.013	1.846*e* − 7	1.459*e* − 5	4.109*e* + 6	0.768
W + Le + F + Ge + Poc	0.013	1.459*e* − 7	1.152*e* − 5	5.200*e* + 6	0.775
W + F + Ge	0.013	9.281*e* − 8	7.332*e* − 6	8.174*e* + 6	0.757
Le + Ss + Ge + Poc	0.013	6.433*e* − 8	5.082*e* − 6	1.179*e* + 7	0.764
W + F + Poc	0.013	5.171*e* − 8	4.085*e* − 6	1.467*e* + 7	0.755
W + Ss + Ge + Poc	0.013	5.068*e* − 8	4.004*e* − 6	1.497*e* + 7	0.763
W + Ss + Poc	0.013	4.817*e* − 8	3.806*e* − 6	1.575*e* + 7	0.754
Le + Ss + Poc	0.013	3.788*e* − 8	2.992*e* − 6	2.003*e* + 7	0.753
W + Ss + Ge	0.013	2.468*e* − 8	1.949*e* − 6	3.074*e* + 7	0.752
Le + Ss + Ge	0.013	2.443*e* − 8	1.930*e* − 6	3.105*e* + 7	0.752
W + F + Ge + Poc	0.013	1.226*e* − 8	9.687*e* − 7	6.186*e* + 7	0.758
W + Le + Ss	0.013	9.055*e* − 9	7.153*e* − 7	8.378*e* + 7	0.748
W + Ss	0.013	9.655*e* − 10	7.628*e* − 8	7.857*e* + 8	0.730
Le + Ss	0.013	3.475*e* − 10	2.745*e* − 8	2.183*e* + 9	0.726
W + Le + Ge	0.013	7.183*e* − 11	5.674*e* − 9	1.056*e* + 10	0.730
W + Le + Ge + Poc	0.013	6.835*e* − 11	5.399*e* − 9	1.110*e* + 10	0.739
W + Le + Poc	0.013	3.995*e* − 11	3.156*e* − 9	1.899*e* + 10	0.727
Le + F + Ge	0.013	3.838*e* − 11	3.032*e* − 9	1.977*e* + 10	0.727
Le + F	0.013	2.344*e* − 11	1.852*e* − 9	3.236*e* + 10	0.715
Le + F + Poc	0.013	8.976*e* − 12	7.091*e* − 10	8.452*e* + 10	0.721
Le + F + Ge + Poc	0.013	6.562*e* − 12	5.184*e* − 10	1.156*e* + 11	0.729
W + Ge	0.013	4.111*e* − 12	3.248*e* − 10	1.845*e* + 11	0.708
W + Ge + Poc	0.013	3.515*e* − 12	2.777*e* − 10	2.158*e* + 11	0.717
W + Le	0.013	2.243*e* − 12	1.772*e* − 10	3.382*e* + 11	0.705
W + Poc	0.013	1.730*e* − 12	1.366*e* − 10	4.386*e* + 11	0.704
W	0.013	9.747*e* − 14	7.701*e* − 12	7.782*e* + 12	0.679
Le + Ge + Poc	0.013	1.394*e* − 14	1.101*e* − 12	5.442*e* + 13	0.693
Le + Ge	0.013	1.326*e* − 14	1.048*e* − 12	5.719*e* + 13	0.682
Ss + F + Ge	0.013	3.208*e* − 15	2.534*e* − 13	2.365*e* + 14	0.687
Ss + F + Ge + Poc	0.013	2.238*e* − 15	1.768*e* − 13	3.389*e* + 14	0.695
Le + Poc	0.013	1.655*e* − 15	1.307*e* − 13	4.584*e* + 14	0.672
Ss + F + Poc	0.013	7.308*e* − 16	5.774*e* − 14	1.038*e* + 15	0.680
Ss + Ge + Poc	0.013	2.201*e* − 16	1.739*e* − 14	3.446*e* + 15	0.674
Ss + F	0.013	1.144*e* − 16	9.036*e* − 15	6.632*e* + 15	0.659
Ss + Ge	0.013	3.897*e* − 17	3.079*e* − 15	1.947*e* + 16	0.654
Le	0.013	3.039*e* − 17	2.400*e* − 15	2.497*e* + 16	0.639
Ss + Poc	0.013	9.778*e* − 18	7.725*e* − 16	7.758*e* + 16	0.647
Ss	0.013	4.226*e* − 21	3.339*e* − 19	1.795*e* + 20	0.590
F + Ge	0.013	1.565*e* − 32	1.237*e* − 30	4.846*e* + 31	0.417
F + Ge + Poc	0.013	5.599*e* − 33	4.424*e* − 31	1.355*e* + 32	0.424
Ge + Poc	0.013	6.897*e* − 36	5.448*e* − 34	1.100*e* + 35	0.346
F + Poc	0.013	6.589*e* − 36	5.206*e* − 34	1.151*e* + 35	0.345
Ge	0.013	1.971*e* − 36	1.557*e* − 34	3.849*e* + 35	0.313
F	0.013	9.958*e* − 37	7.867*e* − 35	7.618*e* + 35	0.306
Poc	0.013	2.300*e* − 41	1.817*e* − 39	3.298*e* + 40	0.188
Null model	0.013	1.435*e* − 46	1.134*e* − 44	5.286*e* + 45	0.000

## Footnotes

1 As the words imply, predictions follow from the prior distribution; postdictions follow from the posterior distribution.

2 Specific information about the data collection can be found on their website http://www.gallup.com/178667/gallup-world-poll-work.aspx. Gallop’s complete report can be downloaded from http://worldhappiness.report/ed/2018/.

3 All JASP commands in the input menu are typeset like this.

4 The model space considered should be predetermined and preferably preregistered before commencing with the analysis. We enlarge the model space here to meet the model assumptions. Strictly speaking, the results should be viewed as exploratory.

5 Although JASP rounds the posterior inclusion probabilities to 1, they never equal 1 exactly.

6 When adjusting the model terms it can be inconvenient that JASP continually updates the results. A trick to disable this is to temporarily remove the dependent variable while adjusting the model terms.

7 The *α* and *β* parameters of the beta-binomial prior can be set individually. Alternatively it is possible to choose the Wilson model prior or the Castillo model prior, which are both variants of the beta-binomial prior (Castillo, Schmidt-Hieber, Van der Vaart, et al., 2015; Wilson, Iversen, Clyde, Schmidler, & Schildkraut, 2010). The Wilson model prior sets *α* = 1 and *β* = *λ**p*, where *p* is the number of predictors in the model and *λ* is a parameter set by the user. The Castillo model prior sets *α* = 1 and *β* = *p*^*u*^, where *p* is the number of predictors in the model and *u* is a parameter set by the user. Both the Wilson and the Castillo prior assign more mass to models with fewer predictors.
